# Genes affecting novel seed constituents in *Limnanthes alba* Benth: transcriptome analysis of developing embryos and a new genetic map of meadowfoam

**DOI:** 10.7717/peerj.915

**Published:** 2015-05-19

**Authors:** Mary B. Slabaugh, Laurel D. Cooper, Venkata K. Kishore, Steven J. Knapp, Jennifer G. Kling

**Affiliations:** 1 Department of Crop and Soil Science, Oregon State University, Corvallis, OR, United States of America; 2 Department of Botany and Plant Pathology, Oregon State University, Corvallis OR, United States of America; 3 Monsanto Company, Bengaluru Area, India; 4 Department of Plant Sciences, University of California-Davis, Davis, CA, United States of America

**Keywords:** Meadowfoam, Desaturase, KCS, LPAT, Glucolimnanthin, Limnanthes

## Abstract

The seed oil of meadowfoam, a new crop in the Limnanthaceae family, is highly enriched in very long chain fatty acids that are desaturated at the Δ5 position. The unusual oil is desirable for cosmetics and innovative industrial applications and the seed meal remaining after oil extraction contains glucolimnanthin, a methoxylated benzylglucosinolate whose degradation products are herbicidal and anti-microbial. Here we describe EST analysis of the developing seed transcriptome that identified major genes involved in biosynthesis and assembly of the seed oil and in glucosinolate metabolic pathways. mRNAs encoding acyl-CoA Δ5 desaturase were notably abundant. The library was searched for simple sequence repeats (SSRs) and single nucleotide polymorphisms (SNPs). Fifty-four new SSR markers and eight candidate gene markers were developed and combined with previously developed SSRs to construct a new genetic map for *Limnanthes alba*. Mapped genes in the lipid biosynthetic pathway encode 3-ketoacyl-CoA synthase (KCS), Δ5 desaturase (Δ5DS), lysophosphatidylacyl-acyl transferase (LPAT), and acyl-CoA diacylglycerol acyl transferase (DGAT). Mapped genes in glucosinolate biosynthetic and degradation pathways encode CYP79A, myrosinase (TGG), and epithiospecifier modifier protein (ESM). The resources developed in this study will further the domestication and improvement of meadowfoam as an oilseed crop.

## Introduction

Meadowfoam (*Limnanthes* sp.) is an herbaceous winter annual native to the West Coast of North America (Mason, 1952). The seed oil is distinctive due to a high content of C20 and C22 fatty acids with Δ5 desaturation (Miller et al., 1964), first characterized in a systematic USDA survey (Earle et al., 1960). Unusual plant fatty acids (FAs) are widespread with more than 300 examples now documented in various species (Aitzetmuller, Matthaus & Friedrich, 2003). Many of these have potential nutritional uses or novel commercial and industrial uses as renewable sources of raw materials. The synthesis of unusual FAs is restricted to developing seed tissues and they accumulate, often to high levels, in seed storage lipid as triacylglycerol (TAG) (Napier, 2007).

The exceptional oxidative stability and lubricity of meadowfoam oil is attributed in part to the proximity of the double bond and the FA carboxy terminus. These properties make it desirable for use in cosmetics and as a source of fatty acids for bio-based applications such as specialized surfactants, lubricants, and plasticizers including derivatives such as estolides and *δ*-lactones (Burg & Kleiman, 1991; Erhan, Kleiman & Isbell, 1993; Isbell, 1997; Wohlman, 2001). Blending of meadowfoam fatty acid methyl esters with soybean-derived biodiesel improved stability, viscosity, and lubricity of the vegetable fuel (Moser, Knothe & Cermak, 2010).

Most plants that accumulate unusual FAs are wild species that are not agronomically adapted. Encouraged by the agricultural potential of certain *Limnanthes* species, however, a domestication and breeding program at Oregon State University established meadowfoam as a rotation crop for grass seed farmers in the Willamette Valley, where cultivars developed from wild accessions of *L. alba* are currently grown for specialized markets (Jolliff et al., 1981; Knapp & Crane, 1999; Knapp, Crane & Brunick, 2005). Meadowfoam is the richest known source of Δ5 monounsaturated FAs and presently the sole commercial source of Δ5-unsaturated very long chain fatty acids (VLCFAs).

Key activities responsible for determining the seed oil phenotype of meadowfoam include a microsomal elongase (KCS) that extends 16:0- and 18:1-acyl-CoA substrates to C20 and C22 VLCFAs, and an unusual desaturase (Δ5DS) that introduces a double bond in >80% of the elongated chains (Pollard & Stumpf, 1980; Moreau, Pollard & Stumpf, 1981). In *L. alba* this produces a fatty acid phenotype of ∼63% 20:1Δ5, ∼4% 22:1Δ5, 10–17% 22:1Δ13 (erucic acid), and 13–19% 22:2Δ5Δ13 (dienoic acid) (Knapp & Crane, 1995). Unlike in most other species in the Brassicales that synthesize VLCFAs, the meadowfoam lysophosphatidyl acyltransferase (LPAT) inserts erucic acid into the *sn*-2 position of TAG (Cao, Oo & Huang, 1990; Laurent & Huang, 1992; Lassner et al., 1995; Brown et al., 2002). The meadowfoam genome has been “mined” for its KCS, Δ5DS, and LPAT genes with the aim of engineering designer lipids in established oilseeds such as rapeseed and soybean (Lassner et al., 1995; Cahoon et al., 2000; Jadhav et al., 2005; Nath et al., 2009). To date none of the transgenic efforts has reproduced the high 20:1Δ5 phenotype of meadowfoam oil, although elevation of erucic acid to 72% in rapeseed transformed with *L. douglasii* LPAT was reported (Nath et al., 2009).

Meadowfoam seeds also contain 3–4% (w/w) glucolimnanthin (Ettlinger & Lundeen, 1956), a phenylalanine-derived 3-methoxybenzyl glucosinolate whose nitrile and isothiocyanate degradation products are toxic to germinating seedlings, fungal pathogens, and insects (Vaughn, Boydston & Mallory-Smith, 1996; Stevens et al., 2009; Zasada et al., 2012). Although the endogenous glucosinolate-degrading enzyme, myrosinase, is inactivated by heat treatment during the oil extraction process, fermenting the residual seed meal with crushed enzyme-active meadowfoam seeds generates breakdown products with pre-emergent herbicidal activity (Stevens et al., 2009). The efficacy of this by-product could be improved by augmenting the glucolimnanthin content of meadowfoam cultivars (Brown & Morra, 2005). A recent survey of meadowfoam accessions revealed a nine-fold variation in seed glucolimnanthin content among 90 *L. alba* breeding lines, indicating substantial genetic diversity in the primary gene pool (Veslasco et al., 2011).

The objectives of the present study were to (i) catalogue lipid biosynthetic and glucosinolate ESTs in the developing seed transcriptome, (ii) recover simple sequence repeat (SSR) and single nucleotide polymorphism (SNP) markers from EST contigs, and (iii) construct a genetic map of *L. alba* populated with SSR markers as well as important candidate genes for lipid synthesis and glucosinolate metabolism. These results can be used to further current meadowfoam breeding goals that include increasing the seed oil content and creating cultivars with altered oil and glucosinolate phenotypes.

## Methods

### Plant materials

Seeds of meadowfoam cultivar MF164 ‘Ross’ (Knapp, Crane & Brunick, 2005), a non-inbred *L. alba* subsp. *alba* population undergoing recurrent selection for oil content, were germinated on moistened blotter paper in the dark at 5 °C for one week, transferred to a growth chamber at 15 °C with 8 h of light/day for two weeks, transplanted to pots for continued growth, then transferred to the greenhouse at 20–21 °C with 16 h light at 7 weeks after planting. Flowers were hand-pollinated and the developing seeds were harvested 14–22 days after flowering. Embryos were excised from seed coats, collected into liquid N_2_, and stored at −80 °C until use.

### RNA extraction and EST library construction

Total RNA was isolated from developing embryos using TRIzol reagent (Gibco-BRL, Grand Island, New York, USA) according to the manufacturer’s instructions and further purified using RNeasy MinElute Cleanup columns (Qiagen, Hilden, Germany). PolyA+ RNA was isolated using the Poly(A) Purist kit (Ambion) according to manufacturer’s instruction. A cDNA library was prepared using the Creator SMART cDNA Library Construction Kit (BD Biosciences, San Jose, California, USA) as directed by the manufacturer’s instructions. cDNAs above ∼500 bp were directionally cloned into Clontech pDNR-LIB vector after SfiI digestion. Gel analysis of a trial transformation showed 100% recombinants with inserts ranging in size from 450 bp to 1.5 kbp and 90% of inserts >500 bp. The ligated library was provided to The Institute for Genomic Research (TIGR, Rockville MD) for electro-transformation and sequencing. Clones were sequenced from the 5′ end of cDNA inserts using the Sanger method and the universal T7 primer (TAATACGACTCACTATAGGG).

### Clustering analysis and annotation

ESTs were cleaned of poor quality and low complexity sequences and trimmed to exclude vector and adaptor sequences. 15,331 EST sequences were deposited in GenBank (http://www.ncbi.nlm.nih.gov/nucest/?term=meadowfoam). Table S1 is a look-up table for converting between the sequence names assigned for GenBank submission and sequence names assigned by TIGR. Cleaned sequences were assembled into 1,352 contigs using CAP3 software. The parameters used for assembly were a minimum of 40 base pair overlap, overlap percent identity cutoff of >94%, and a maximum unmatched overhang of 30 base pairs. Contigs were assigned names TC1…TC1352. The EST sequences contained in each contig are listed in Table S2. Assembled contigs and singletons were compared to a variety of protein databases using blastx.

### SSR marker development and genotyping

Unigenes from the EST analysis were screened for simple sequence repeats (EST-SSRs) with a minimum repeat size of six for dinucleotide repeats and five for tri-, tetra, and hexanucleotide repeats. Repeat sequences with di-, tri-, and tetranucleotide SSRs were also mined from trial methyl-filtered and unfiltered genomic libraries prepared from meadowfoam leaf DNA and analyzed by Orion Genomics (St. Louis, Missouri, USA) (genomic-SSRs). Primer3 software was used to pick 172 primer sets (110 EST-SSRs and 62 genomic-SSRs) designed to yield 150–400 bp PCR products. Primers had a Tm of ∼60 °C, and GC content between 40 and 60%. Forward primers were labeled with either HEX or FAM fluorescent tags. Primer sequences are available in Table S3.

### Linkage map construction

The linkage map was built using an inter-subspecific backcross between MF40-11 (*L. alba* subsp. *alba*) and MF64 (*L. alba* subsp. *versicolor*). Parents were inbred to S_5_ prior to being crossed. SSRs and candidate gene markers were genotyped on 90 BC_1_ progeny of the [(MF40-11 × MF64) × MF64] cross. SSR genotyping was as described (Kishore et al., 2004) except that fragments were separated on an ABI Prism 3,100 capillary system (Applied Biosystems, Carlsbad, California, USA) at the Central Services Lab, Oregon State University, and scored using Genotyper software and manual evaluation. Candidate genes were scored as described below. Linkage maps were assembled using JoinMap 4 software with BC1 population type codes (Van Ooijen, 2006). The input dataset contained genotypes for 123 SSR markers and eight candidate genes. Markers were assigned to five linkage groups using a test for independence LOD score of 6.0, and ordered using the regression mapping algorithm with a recombination frequency threshold of 0.40, a LOD threshold larger than 1.00, and a jump threshold value of 5.0. The Haldane mapping function was used to translate recombination frequencies into map distances.

### SNP discovery

The program AutoSNP (Barker et al., 2003) was used to search for putative SNP sites within contigs. An independent CAP3 analysis, done with similar parameters used for the TIGR CAP3 analysis, yielded 1,376 contigs. AutoSNP parameters were set to report SNP positions in contigs with minor allele frequencies of at least one for contigs with 2–4 ESTs, two for contigs with 5–6 ESTs, three for contigs with 7–9 ESTs, four for contigs with 10–12 ESTs, five for contigs with 13–15 ESTs, and six for contigs with 16 + ESTs.

### Genotyping assays for selected candidate genes

For each meadowfoam, contig of interest homologous genomic and cDNA sequence(s) from Arabidopsis and other plants were retrieved from databases and sequences were aligned using ClustalW to identify putative intron locations. Multiple PCR primer sets were designed to produce intron-containing PCR products and tested on MF40-11 and MF64 parental templates and a pooled DNA sample from 12 BC_1_ progeny. The resulting PCR products were screened for length polymorphisms on agarose gels and for single strand conformational polymorphisms (SSCP) on silver-stained polyacrylamide gels as described (Slabaugh et al., 1997). SSCP analyses can resolve DNA segments that differ in a single nucleotide (Spinardi, Mazars & Theillet, 1991) and typically produce two SSCP bands per PCR product. Preliminary trials were scored for number of loci amplified, polymorphism between parents, and transfer of polymorphic loci to progeny. The primer sequences selected for marker generation are provided in Table S4.

**KCS11**. Fourteen ESTs comprising Contig 248 were aligned with *L. douglasii* FAE cDNA sequence AF247134. Primers KCS1F and KCS4R were designed from sequence segments with no SNPs among the contig ESTs. The 460-bp PCR product indicated absence of an intron in the KCS gene.

Δ **5DS**. ESTs representing all eight haplotypes comprising Contig 104 were aligned with Arabidopsis genomic sequences At1g06080 (ADS1) and At3g15870 (ADS3), their respective cDNAs, and *L. douglasii* Δ5DS cDNA AF247133. This identified four putative intron locations in the meadowfoam genes. Primers DS218F and DS219R were designed to flank both sets of (CT)_*n*_ repeats in the 5′-UTR and anneal to all haplotypes. Primers DS221F and DS5R matched invariant segments of the Contig 104 sequence and were designed to flank intron 1. These primers produced PCR products of ∼700 bp and ∼750 bp, respectively from MF64 and MF40-11 templates indicating intron 1 sizes of ∼600 and ∼650 in parental DNAs. The intron length polymorphism was used to score segregation of Δ5DS on an agarose gel.

**LPAT2**. Contig 909 was aligned with genomic LPAT sequences from *L. douglasii* (DQ402047) and *L. floccosa* (AF212042), and cDNA sequences from *L. douglasii* (Z46836) and *L. alba* (U32988), which identified four intron locations. Primers LPAT432F and LPAT893R were chosen to include introns 2, 3, and 4 and produced 710-bp PCR products from parental DNAs. The MF40-11 product was resolved by SSCP analysis into two sets of bands but only one set was observed in BC_1_ progeny, indicating heterozygosity in the MF40-11 parent. The transmitted MF40-11 allele was polymorphic with the allele from MF64.

**DGAT2**. Contig 384 was aligned with Arabidopsis genomic sequence At3g51520 (DGAT2) and its cDNA to identify the putative locations of seven introns. Primers DGAT3F and DGAT4R were designed to include intron 4. The 290-bp PCR product indicated a size of ∼100 bp for intron 4 in *L*. *alba*. A strong set and a weak set of co-segregating polymorphic bands were amplified from each parental DNA.

**TGG**. Contig 572 was aligned with Arabidopsis genomic sequences At5g26000 and At5g25980 (TGG1 and TGG2, respectively) and their corresponding cDNAs, to identify the putative locations of 11 introns. Primers TGG436F and TGG792R were designed to include introns 8 through 11. The 760-bp PCR fragment indicated that a total of ∼400 bp of intronic sequence was included in *Limnanthes* products. The primers produced three sets of SSCP bands from each parent and two complex but co-segregating patterns in BC_1_ progeny, indicating a small gene family.

**ESM1,2,3**. Contigs 183 and 184 were aligned with Arabidopsis ESM1 genomic (At3g14210) and corresponding cDNA sequences to identify four intron positions. Primers ESM-5F (upstream of intron 2, matches Contigs 183 and 184), ESM-2R (downstream of intron 3, Contig 183-specific) and ESM-4R (downstream of intron 3, Contig 184-specific) were used. SSCP analysis showed that primers 5F and 2R (Contig 183-specific) amplified three co-segregating loci; primers 5F and 4R (Contig 184-specific) amplified one monomorphic locus.

**CYP79A**. Amino acid sequences from cytochrome P450 genes CYP79A (Phe substrate), CYP79B (Trp substrate), and CYP79E (Tyr substrate) from *Sinapis alba*, *B. napus*, sorghum, arrowgrass, and Arabidopsis were collected from databases and aligned to identify residues specifically conserved in each CYP79 subclass. Degenerate primers were designed to amplify the subgroups. Primer combinations were trialed on parental DNA and the 12-progeny BC_1_ bulk using a touch-down PCR protocol in which the annealing temperature was gradually decreased from 55 C to 50 C. Primers CYP203F (motif EEIEHV[D/E]) and CYP211R (motif HPVAPFN), targeted to the CYP79A class, produced a pair of polymorphic bands of the expected size. Primers CYP201F (motif EHMEAMF) and CYP217R (motif QESDIPKL), targeted to the CYP79B class, produced a pair of monomorphic bands of the expected size.

## Results and Discussion

### Construction and annotation of a developing embryo cDNA library

To identify genes important for meadowfoam seed phenotypes, a cDNA library was made from developing embryos of *L.alba* subsp. *alba* excised from their seed coats 14–22 days after fertilization (DAF). Our intent was to sample developmental stages that included plastidial fatty acid synthesis, cytoplasmic acyl-chain modification and storage lipid biosynthesis, as well as glucosinolate accumulation. Embryos were collected from 20 plants of cultivar ‘Ross,’ a heterogeneous, open-pollinated population undergoing recurrent selection for desirable agronomic traits (Knapp, Crane & Brunick, 2005). mRNA was isolated from a heterogeneous population to capture sequence variation that could be utilized for DNA-based markers.

Sanger-sequenced cDNAs had an average read length >500 bp. After cleaning, 12,652 of the 15,331 high-quality reads were assembled into 1,352 contigs (Table 1). The number of ESTs per contig ranged from 2 to 460. Thirty-seven per cent of the sequences were assigned to 1,258 contigs that contained from 2 to 20 ESTs and 45.5% were assigned to 94 large contigs with >20 ESTs. Most of the large contigs encoded either 2S or 12S seed storage protein precursors. Sixty-two contigs were annotated as most similar to the 2S seed storage protein mabinlin from *Capparis masaikai*, a species in a sister family to Limnanthaceae within the order Brassicales. Mabinlin is of interest due to an amino acid motif in the protein that interacts with the sweet taste receptor (Li et al., 2008). The *L. alba* homologues, however, contained only part of this motif. As expected in an oilseed, ESTs encoding proteins with structural roles in lipid synthesis, transport, and storage such as acyl carrier protein (ACP, 34 ESTs), acyl-CoA binding protein (ACBP, 216 ESTs), and oleosin (OLE, 267 ESTs) were well represented and comprised in aggregate 3.4% of the ESTs whereas ESTs annotated as encoding enzymes of lipid metabolism represented only 1.2% of the 14–22 DAF transcriptome. Comparison of the meadowfoam EST unigenes against Arabidopsis proteins revealed 70% hits with E-values ≤10^−5^.

**Table 1:** CAP3^a^ assembly statistics for meadowfoam developing seed cDNA library.

Description	Number	Percentage
Total number of EST sequences	17,376	100%
Number of high-quality sequences	15,300	88%
Number of contigs	1,352	82% of high-quality sequences
Number of singletons	2,679	18% of high-quality sequences
Number of unigenes	4,031	
Average length of EST	528 bp	

**Notes.**

a CAP3 parameters: % identity >94, minimum 40 bp overlap, maximum 30 bp overhang.

### SSR markers mined from cDNA and genomic libraries

The EST unigene sequences and an additional 2,864 sequences from pilot-scale methyl-filtered and unfiltered genomic libraries prepared from *L. alba* subsp. *versicolor* (M Slabaugh, 2004, unpublished data) were searched for simple sequence repeats suitable for marker development. These searches produced 178 EST library-derived and 80 genomic library-derived candidates with repeat units of 2, 3, 4, or 6, for an overall frequency of 4.4% and 2.8%, respectively (Table 2). Dinucleotide repeats predominated in both collections but differed in type: the majority of dinucleotide SSRs from ESTs were AG/CT repeats, whereas 84% of the dinucleotide repeats in the genomic libraries were AT repeats. SSRs from genomic sequences had approximately twice the mean number of repeats as those from EST-SSRs: 16.5 vs. 7.5 for dinucleotide repeats and 9.9 vs. 5.6 for trinucleotide repeats. Detailed information regarding 172 candidate SSR loci for which primers could be designed, including primer sequences and allele sizes from multiple germplasm sources, are contained in Tables S3 and S5. These new SSR loci add to the 624 unique SSR loci mined from genomic libraries and described by Kishore et al. (2004).

**Table 2:** Simple sequence repeats (SSRs) in developing meadowfoam embryo EST and genomic DNA sequences.

		Repeat unit length				
		Total	*n* = 2	*n* = 3	*n* = 4	*n* = 6
EST-SSRs^a^	Number detected	178	99	67	4	8
	Primers made	110	54	47	4	5
	Number mapped	44	26	13	2	3
Genomic-SSRs^b^	Number detected	80	68	10	2	0
	Primers made	62	50	10	2	0
	Number mapped	10	7	3	0	0

**Notes.**

a 4,031 EST unigene sequences were examined.

b 2,864 sequences from genomic libraries were examined.

The functional utility of SSRs gleaned from the EST library was substantially greater than SSRs retrieved from genomic libraries as 40% of the 110 EST-SSR candidates but only 16% of the 62 genomic-SSR candidates were polymorphic in our mapping population (Table 2). Overall, 32% of dinucleotide-SSR primers and 28% of trinucleotide-SSR primers produced scoreable SSRs. Thirty-eight percent of the candidate PCR products were not polymorphic, and remaining primers either failed to amplify parental DNA (23%) or produced ambiguous products (7%).

### SNP polymorphisms in the EST unigene set

*L. alba* subsp. *alba* EST contigs were analyzed for the presence of single nucleotide polymorphisms (SNPs) using AutoSNP software (Barker et al., 2003). When run with stringent minimum redundancy requirements (see ‘Materials and Methods’) AutoSNP detected 7,193 SNPs in 1,376 contigs of mean length 684 nt, yielding an average frequency estimate of 1 SNP per 131 nt of contig sequence. These calculations, however, included contigs with few ESTs where SNP variants may be under-represented or under-scored due to redundancy requirements, and those with >40 ESTs where SNP calls were exceptionally high, likely due to paralog pooling by the CAP3 analysis. Restricting the analysis to contigs with >4 and <41 ESTs (31% of the contig set) produced an estimate of 1 SNP per 294 nts in the out-crossing *L. alba* breeding population we sampled. The identified SNP sites can be used to develop additional markers for the genetic map as well as enable design of SNP markers for candidate genes of interest in targeted breeding projects. AutoSNP results, including sequence alignments and tabulated SNP sites, are available as HTML files from the corresponding author.

### Construction of an inter-subspecific meadowfoam genetic map

**Figure 1:**
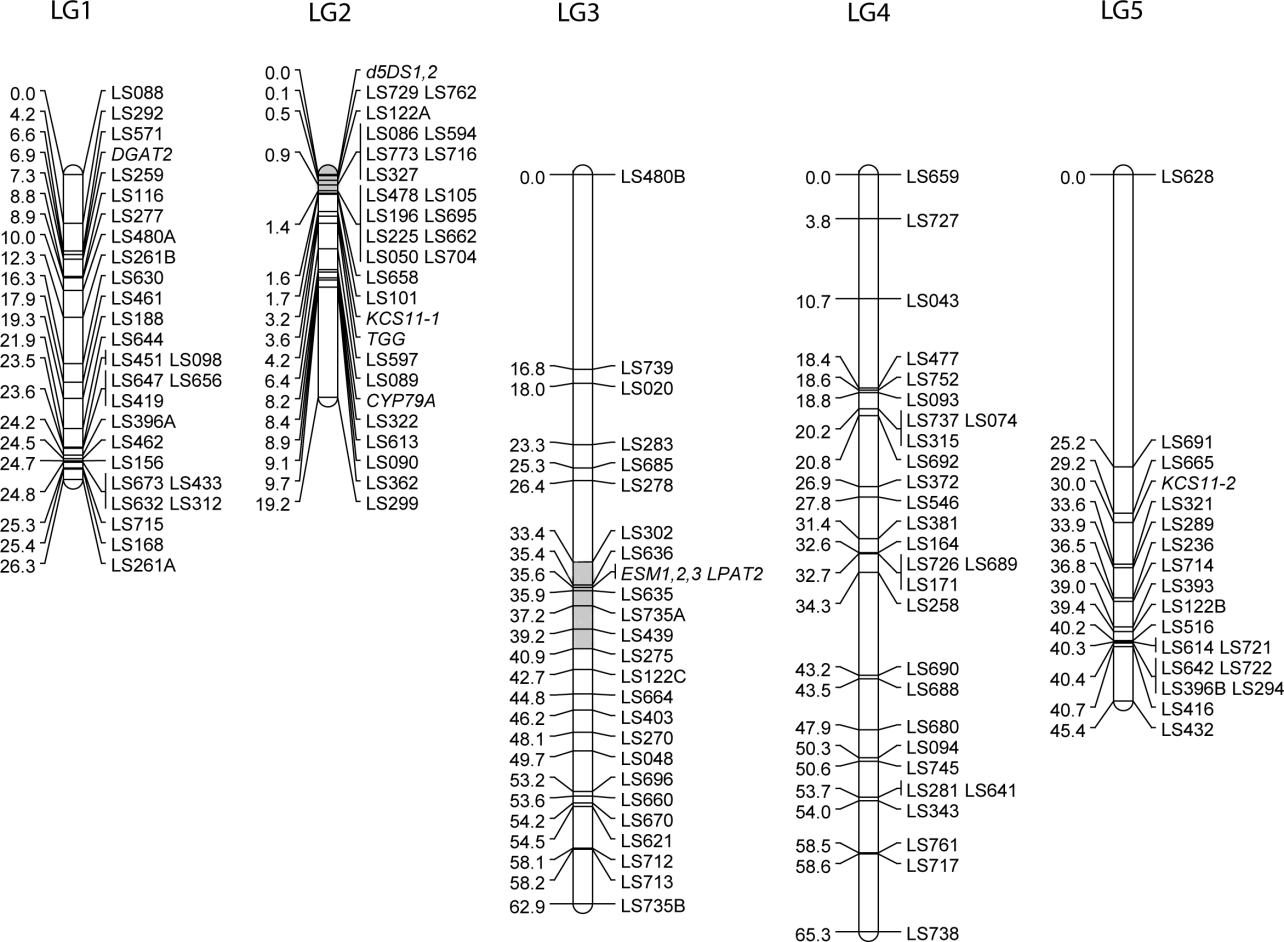
The genetic linkage map of *Limnanthes alba.* Five linkage groups (LG) were constructed from a BC1 mapping population of 90 individuals derived from an intersubspecific cross between MF40-11 (*L. alba* subsp. *alba*) and MF64 (*L. alba* subsp. *versicolor*). SSR markers developed from EST and genomic sequences are shown with LS prefixes, and candidate gene markers genotyped using sequence-specific PCR/SSCP assays are shown in italics. Regions of LG2 and LG3 previously identified by QTL analysis (Gandhi et al., 2009) as harboring genetic factors affecting erucic acid levels are shaded light grey.

Previous maps for meadowfoam used non-transferable dominant AFLP markers (Katengam et al., 2001) or were constructed specifically for QTL studies of certain traits (Gandhi et al., 2009). To develop a framework genetic map populated with easily assayed, highly polymorphic markers, we used a BC_1_ population developed from a cross between partially inbred (S_5_) individuals from *L. alba* subsp. *alba* (MF40-11) and *L. alba* subsp. *versicolor* (MF64, recurrent parent). Ninety backcross progeny were genotyped for 54 SSR markers developed during the present study and 74 previously-developed genomic markers (Kishore et al., 2004). The SSR loci grouped into five linkage groups presumably corresponding to the five haploid chromosomes of this species. Eight candidate gene loci were added to the map by genotyping the BC_1_ population with sequence-specific PCR products whose segregating alleles could be distinguished by single strand conformational polymorphism (SSCP) (Spinardi, Mazars & Theillet, 1991). SSCP polymorphisms used to map candidate genes are shown in Fig. S1 and primer sequences are listed in Table S4. The map assembled by JoinMap 4 was 219 cM long with 19–29 loci per linkage group (Fig. 1). The groupings were stable to LOD 10, except for LG5 where the distal marker LS628 was lost at LOD 9. The longest gap was 25.2 cM on linkage group 5. Segregation ratios for 37 loci on linkage groups 3 and 4 were significantly distorted (*p* ≤ 0.05). All of the distorted loci had an excess of MF40-11 alleles suggesting selection for heterozygous genotypes. Distortion occurred in the middle of linkage group 3 whereas distortion on linkage group 4 encompassed only the upper half of the linkage group.

The new map was co-linear with a previously published genetic map constructed from an independently-generated intra-subspecific *L. alba* subsp. *alba* F_2_ population (Gandhi et al., 2009) as determined by genotyping 20 of the markers from the F_2_ map that were also polymorphic in the inter-subspecific BC_1_ population of this study. The common loci mapped in the same order except for a minor rearrangement of two closely-spaced markers on linkage group 1 and co-segregation of two markers on linkage group 2 in the BC_1_ map that were 7 cM apart on the F_2_ map. These common markers permitted naming and orientation of linkage groups to correspond to Gandhi et al. (2009), except for linkage group 5 that had only one marker in common. Side by side comparisons indicated that each map contained markers in telomeric regions not covered by the other (not shown).

### Developing seed transcriptome

Table 3 catalogs our analysis of the 14–22 DAF seed transcriptome for lipid and glucosinolate metabolism ESTs. ESTs encoding enzymes involved in fatty acid synthesis in the plastid (ACCase, KAS I, KAS II, ER, HAD, KAR) and fatty acid elongation in the ER (ACCase, KCS, ECR, KCR) were each present at low but roughly stoichiometric levels (0.02–0.09% of total ESTs). ESTs for the structural protein involved in conveyance of nascent acyl-CoA chains from the plastid to the ER, acyl-CoA-binding protein (ACBP), and the surface protein that encapsulates oil droplets, oleosin (OLE), were 30- to 40-fold more prevalent than those for any of the enzymatic activities. The number of haplotypes detected for enzyme-ESTs was one or two except for the elongase KCS11 (5 haplotypes) and ADS3-like Δ5DS (8 haplotypes). Multi-gene families for these activities were confirmed by subsequent mapping experiments (see below). ESTs for the cytoplasmic ADS3-like Δ5DS were abundant whereas only two ESTs were detected for stearoyl-ACP desaturase, the plastidial enzyme that produces 18:1Δ9. Missing entirely from the meadowfoam EST collection were transcripts encoding the plastidial thioesterases FATA and FATB, and the initial TAG assembly enzyme glycerol-phosphate acyl-CoA transferase, GPAT. However, the low prevalence of ESTs for thioesterases and GPAT as quantitated by deep transcriptional profiling of non-normalized libraries from four other developing oilseeds at a similar developmental stage (Troncoso-Ponce et al., 2011) suggests that these ESTs were likely below detection in our study.

**Table 3:** Seed lipid and glucosinolate metabolism transcripts from the developing embryo EST library.

Gene name(s), following Arabidopsis	*L. alba* contig(s) or singleton(s)	Activity identified by BLASTx	No. of ESTs	Contig size (bp)	No. of SNPs	No. of haplo-types	Representative at gene model
	Lipid synthesis and TAG assembly						
WRI1	Contig 1095	Transcription factor of the AP2/ERWEBP class	2	780	3	2	At3g54320
CAC1, BCCP2	Contig 434	Biotin carboxyl carrier protein	7	1,064	0	1	At5g15530
CAC2, BC	Contig 311	Biotin carboxylase subunit of Het-ACCase	11	531	0	1	At5g35360
KAS I	Contig 418	3-Ketoacyl-ACP synthase I	7	1,110	0	1	At5g46290
KAS II	Contig 555	3-Ketoacyl-ACP synthase II	5	854	4	2	At1g74960
ER	Contig 535	Enoyl-ACP reductase	5	1,253	0	1	At2g05990
HAD	Contig 457	Hydroxyacyl-ACP dehydratase	6	810	5	2	At5g10160
KAR	Contig 803^a^ Contig 1261^a^	3-Ketoacyl-ACP reductase (KAR)	3 2	474 658	9 2	2 2	At1g24360
FAB2/DES	MF1BL10TV MF1CR48TV	Stearoyl-ACP desaturase	2	NA^2^	NA^2^	NA^2^	At2g43710
ADS3	MF1EJ57TV	Palmitoyl-monogalactosyldiacyl glycerol desaturase, delta9 FADS-like	1	NA^2^	NA^2^	NA^2^	At3g15850
FAD8	MF1CZ39TV	Delta-15 desaturase, plastid	1	NA^2^	NA^2^	NA^2^	At5g05580
ACP	Contig 141	Acyl carrier protein	34	787	9	4	At1g54580
LACS4	MF1CK48TV	Long-chain acyl-CoA synthetase	1	NA^2^	NA^2^	NA^2^	At4g23850
ACC1	Contig 1339	Homomeric Acetyl-CoA carboxylase	2	697	3	2	At1g36160
KCS11	Contig 248	3-Ketoacyl-CoA synthase	14	846	16	5	At2g26640
ECR	Contig 567	Enoyl-CoA reductase	5	1,232	8	2	At3g55360
KCR	Contig 405	3-Keto acyl-CoA reductase	8	1,194	8	2	At1g67730
ADS3-like	Contig 104	Delta-5 acyl-CoA desaturase	91	1,411	27	8	At3g15850
FAD2	MF1AB25TVB	Delta-12 fatty acid desaturase, ER	1	NA^2^	NA^2^	NA^2^	At3g12120
ACBP6	Contig 99	Acyl-CoA-binding protein	53	587	0	1	At1g31812
ACBP6	Contig 100	Acyl-CoA-binding protein	37	574	0	1	At1g31812
ACBP6	Contig 101	Acyl-CoA-binding protein	126	585	8	8	At1g31812
LPAT2	Contig 909	Lysophosphatidyl acyltransferase	2	814	7	2	
DGAT2	Contig 384	Diacylglycerol acyltransferase	8	1,156	12	2	At3g51520
LPEAT	Contig 980	Phospholipid/glycerol acyltransferase	2	780	0	1	At1g80950
LTP4	Contig 1122	Lipid transfer protein 4	2	788	9	2	At5g59310
OLE2	Contig 108	Oleosin	86	870	25	6	At5g40420
	Contig 110	Oleosin	2	757	11	2	At2g25890
	Contig 111	Oleosin	78	839	19	4	At2g25890
	Contig 118	Oleosin	5	735	0	1	At3g01570
	Contig 119	Oleosin	22	737	0	1	At3g01570
	Contig 120	Oleosin	22	728	5	3	At3g01570
OLE1	Contig 125	Oleosin	44	746	4	3	At4g25140
	Contig 300, Contig 301	Oleosin	8	647	0	1	At3g01570
	Glucosinolate metabolism						
ESM1	Contig 183	Epithiospecifier modifier/myrosinase-associated protein	5	851	5	3	At3g14210
ESM1	Contig 184	Epithiospecifier modifier/myrosinase-associated protein	15	1,270	15	3	At3g14210
TGG1	Contig 572	*β*-Thioglucoside glucohydrolase/ myrosinase	4	1,098	3	2	At5g26000
MBP	Contig 901	Myrosinase binding protein	3	1,049	NA^c^	NA^c^	At1g52030

**Notes.**

a Non-overlapping contigs: Contig 1,261 covered 5′ end of KAR cds (60%) and contig 803 covered 3′ end of KAR cds (25%).

b NA = Single EST or two non-overlapping ESTs,

c Probable paralogs.

### Meadowfoam seed-expressed fatty acid elongase is a homologue of AtKCS11

More than 98% of the fatty acids in meadowfoam oil are C20 and C22 VLCFAs resulting from elongation of saturated and Δ9-monounsaturated acyl-CoA substrates by ketoacyl-CoA synthase (Pollard & Stumpf, 1980; Sandager & Stymne, 2000). Contig 248 (14 ESTs) encoded an elongase with 76% identity to AtKCS11 (At2g26640), a member of the *ζ*-clade of the Arabidopsis KCS gene family (Joubes et al., 2008). Contig 248 was therefore designated LaKCS11. The deduced amino acid sequence of Contig 248 was 99% identical to an *L. douglasii* cDNA previously designated LdFAE1 (Cahoon et al., 2000). Contig 248 plus a singleton EST (MF1E193TV) together covered ca. 90% of the LaKCS coding sequence. The contig comprised five haplotypes with at least two representatives each, plus two singleton haplotypes, based on 12 SNPs in the cds and 3′ UTR. Cds SNPs were synonymous substitutions except for one conservative codon change, resulting in two polypeptide variants: 12 ESTs encoded Val and 2 ESTs encoded Ile at residue 462 (numbering based on the LdFAE1 sequence).

To map LaKCS11 we amplified genomic DNA with primers that flanked eight SNP sites within the cds. Analysis revealed as many as eight interleaved SSCP bands in the BC_1_ progeny. The segregation patterns were de-convoluted, revealing that the *L. alba* subsp. *alba* and *L. alba* subsp. *versicolor* parents carried contrasting alleles at two unlinked KCS loci. LaKCS11-1 was mapped to LG2 and LaKCS11-2 was mapped to LG5 (Fig. 1). Because the KCS family in Arabidopsis includes 21 genes with functions in various metabolic pathways and differing substrate preferences (Blacklock & Jaworski, 2002; Blacklock & Jaworski, 2006; Joubes et al., 2008), our results raised the question of whether the primers we used might have amplified KCS paralogs in addition to KCS11, if these exist in *Limnanthes*. To address this, we aligned amino acid sequences representing each of the eight Arabidopsis clades with KCS sequences from *Brassica* sp., *Teesdalia*, *Crambe*, *Lunaria*, *Tropaeolium*, and *Limnanthes*. This revealed that the upstream primer we used was in fact located in highly conserved sequence but the downstream primer was targeted to a region that varied extensively between clades and included a *ζ*-clade-specific Phe codon at its 3′ end. We concluded that both LaKCS loci were likely members of the *ζ*-clade.

A major determinant of the VLCFA profile in seeds is the substrate range and activity of the elongase system(s) (Jasinski et al., 2012). Based on biochemical experiments Sandager & Stymne (2000) suggested that in meadowfoam, one system with specificity for saturated substrates elongates to C20 and another system with specificity for mono-unsaturated substrates elongates to C22. However, when Jadhav et al. (2005) co-expressed *L. douglasii* FAE1(LdKCS11) and Δ5DS cDNAs in soybean seeds, the resulting fatty acid profile included 10.6% 20:1Δ5 and 10.0% 20:1Δ11, evidence that a single meadowfoam KCS could extend both saturated and mono-unsaturated substrates. Consistent with this, an embryo-expressed *ζ*-clade KCS from nasturtium (*Tropaeolum majus*) displayed broad substrate activity when transformed into Arabidopsis and tobacco (Mietkiewska et al., 2004) and a recombinant AtKCS11 polypeptide was shown to have elongase activity on both saturated and mono-unsaturated C16–C20 substrates *in vitro* (Blacklock & Jaworski, 2006). We cannot rule out the possibility that the Val/Ile variants at residue 462 in our EST collection might have somewhat different substrate preferences, however, as residues in this part of the protein are predicted to be near the substrate pocket based on modeling (Joubes et al., 2008; Jasinski et al., 2012).

### *L. alba* Δ5DS is highly expressed in mid-development embryos

Contig 104 (91 ESTs) encoded the enzyme that confers unique chemical properties to meadowfoam oil, desaturation at the Δ5 position (Pollard & Stumpf, 1980; Moreau, Pollard & Stumpf, 1981; Cahoon et al., 2000). The abundance of Δ5DS transcripts in the cDNA library likely explains how <20% of fatty acid chains escape Δ5 desaturation in meadowfoam seeds. Δ5DS ESTs grouped into two classes distinguished by either (CT)_6_ (34 ESTs) or (CT)_7_ (57 ESTs) dinucleotide repeats in the 5′-UTR, and nine SNP sites within the cds. Deduced amino acid sequences, however, were invariant. A second (CT)_*n*_ repeat also within the 5′-UTR varied from 8 to 16 repeats in the (CT)_6_ class (5 haplotypes) and 12–14 repeats in the (CT)_7_ class (3 haplotypes), with a single variant accounting for ca. 70% of the sequences in each class. One explanation for this diversity of EST haplotypes is that the *L. alba* genome contains two paralogous Δ5DS genes characterized by (CT)_6_ vs. (CT)_7_ repeats and that diversity in the second repeat motif is attributable to allelic polymorphism among the outbred plants sampled for the cDNA library. However, this explanation was not supported by amplifying DNA from four partially inbred (S_5_) mapping parents in our breeding program with (CT)_6_-specific or (CT)_7_-specific PCR primers, as the results indicated that each line harbored only one or the other of the two classes (not shown).

The presence of at least two Δ5DS genes in the meadowfoam genome was deduced by using primers matching invariant sequences flanking both sets of repeats. When applied to MF40-11 and MF64 parental DNAs these primers produced three sets of polymorphic SSCP bands from each template (Fig. S1). BC_1_ progeny, however, produced only one band set from each parent, suggesting that both parents carried two Δ5DS loci but were each heterozygous at one locus. The co-segregating Δ5DS loci were mapped to the upper end of LG2 (Fig. 1). In sum, our results indicated that the Δ5DS gene family consists of two closely-linked loci that are hyperpolymorphic within populations due to the presence of tandom (CT)_*n*_ repeats in the 5′-UTR.

Gandhi et al. (2009) mapped meadowfoam QTL affecting erucic acid (22:1Δ13) and dienoic acid (22:2Δ5Δ13) in an *L. alba* subsp. *alba* F_2_ population in which factors that promoted a 3- to 4-fold reduction of erucic acid (to ∼3%) were segregating. Erucic and dienoic QTL associated with 22% of the phenotypic variance were coincident and had opposite effects on erucic and dienoic levels. These QTL mapped to LG2 (see Fig. 3 of Gandhi et al., 2009). As estimated from four common markers on LG2, the Δ5DS EST loci mapped in the present study are within the erucic/dienoic QTL peak of the F_2_ map (shaded in Fig. 1), lending support to the suggestion that the Δ5DS is a candidate gene underlying the low erucic phenotype. Because erucic and dienoic acids are presumed to have a precursor-product relationship, differing expression levels, specific activity, or substrate preference in this enzyme could contribute to oil phenotype differences, as well as explain the coincidence of erucic and dienoic QTL on LG2.

The Δ5DS’s encoded by *L. alba* and *L. douglasii* (Cahoon et al., 2000) are members of the large “Delta9-FADS-like” or ADS subfamily found in eukaryotes including vertebrates, insects, higher plants and fungi, as well as a wide range of bacteria. These non-heme, iron-containing, ER membrane-bound enzymes exhibit a diversity of subcellular localization, lipid substrate utilization, regiospecificity, and biological function (see http://www.ncbi.nlm.nih.gov/Structure/cdd/cddsrv.cgi?uid=58171). Blastp revealed that *L. alba* and *L. douglasii* Δ5DS s, which lack a chloroplastic transit peptide, are 98% identical to each other and show ∼60% identity to a large number of proteins including palmitoyl-monogalactosyldiacylglycerol Δ7DSs similar to the chloroplastic ADS3 from Arabidopsis (Heilmann et al., 2004), predicted cytoplasmic Δ9DSs from many plant species, and two Δ5DS s from *Anemone leveillei* (Sayanova et al., 2007). The acyl-lipid substrate for the Δ5DS s remains to be proven, although the preferred substrates for the meadowfoam Δ5DS are putatively acyl-CoAs, as inferred from biochemical time course assays using a variety of substrates and cell free extracts of developing seeds (Moreau, Pollard & Stumpf, 1981). Sayanova et al. (2007) examined the acyl-CoA pools in developing seeds of Arabidopsis transformed with two *A. leveillei* Δ5DS cDNAs and found evidence that both enzymes use acyl-CoA substrates.

### Genes for LPAT and DGAT

ESTs for two activities in the acyl-CoA dependent Kennedy pathway for TAG assembly were present in the meadowfoam library. Contig 909, comprising two overlapping ESTs covering the 3′ third of the cds, was annotated as lysophosphatidyl acyltransferase, homologous to endoplasmic reticulum-based LPATs from other plant species and referred to as LPAT2 in most oilseeds (Taylor et al., 2010). Segregation analysis of SSCP patterns (Fig. S1) indicated the presence of a single LPAT2 locus that mapped to the interior of LG3 (Fig. 1). A QTL for erucic acid associated with 13% of the phenotypic variability was previously mapped (Gandhi et al., 2009) to the corresponding region of LG3 (shaded in Fig. 1), as estimated from three SSR markers in common between the maps. Although *L. douglasii* LPAT2 showed a preference for 22:1-acyl-CoA substrate *in vitro*, the enzyme exhibited a broad substrate range (Brown et al., 2002), and it is feasible that variation at the erucic-inserting LPAT2 locus could underly phenotypic variability in erucic acid in meadowfoam seed oil.

The final enzyme in TAG synthesis via the Kennedy pathway, acyl-CoA diacylglycerol acyltransferase, or DGAT, was encoded by Contig 384. Meadowfoam DGAT2 ESTs displayed two haplotypes among eight ESTs and we mapped the DGAT2 locus to the upper arm of LG1. The lack of a DGAT1 homologue in our library is consistent with suggestions that DGAT2 functions in seed-specific TAG biosynthesis and may be preferentially involved in transfer of unusual fatty acids into storage oil (Shockey et al., 2006; Banilas et al., 2011). Work in several plant species suggests that DGAT activity is rate-limiting in TAG biosynthesis and thus has an effect on final seed oil content, as shown in Arabidopsis (Jako et al., 2001), *B. napus* (Weselake et al., 2008), soybean (Lardizabal et al., 2008), and maize (Zheng et al., 2008).

### Mapping genes in meadowfoam glucosinolate metabolism

Myrosinase, a glucoside glucohydrolase that initiates the degradation of glucosinolates upon tissue disruption, was encoded by Contig 572, a homologue of Arabidopsis TGG2 (At5g26000). Two haplotypes were present among the four ESTs, and three sets of SSCP bands from parental DNAs (MF40-11 and MF64) and BC_1_ progeny indicated a small multi-gene family. The myrosinase genes were localized to the upper end of LG2 (Fig. 1).

Contigs 183 and 184 were homologous to an Arabidopsis gene, EPITHIOSPECIFIER MODIFIER1 (ESM1, At3g14210), that directs the glucosinolate hydrolysis pathway towards isothiocyanate breakdown products (Zhang, Ober & Kliebenstein, 2006). Contigs 183 and 184 likely represent paralogous ESM1 genes as there were eight amino acid differences between the deduced protein sequences. Contig 183-specific primers amplified three polymorphic sequences from the mapping parent DNAs (Fig. S1), and LaESM1,2,3 genes were localized to the interior of LG3, co-segregating with LPAT2 (Fig. 1). Contig 184-specific primers produced a single set of non-polymorphic bands from each parent.

Glucosinolates are synthesized in maternal vegetative tissues and actively translocated into developing seeds (Lykkesfeldt & Moller, 1993; Nour-Eldin et al., 2012). Consistent with this we did not detect transcripts for glucosinolate biosynthetic enzymes in the meadowfoam developing embryo library. However, because enhancing seed glucosinolate content is a goal of meadowfoam breeding (Veslasco et al., 2011), we developed a marker for CYP79A, the gene encoding the cytochrome P450 enzyme that catalyzes the rate-limiting step in benzyl glucosinolate synthesis from phenylalanine (Wittstock & Halkier, 2000). Primers designed to exclusively produce an intron-containing CYP79A gene fragment generated a single polymorphic product that was localized to LG2 (Fig. 1).

## Conclusions

Transcriptome analysis of meadowfoam embryos identified key genes essential to synthesis of the unusual VLCFAs characteristic of *Limnanthes* seeds and indicated that the prevalence of Δ5 desaturation in meadowfoam TAG is due to an abundance of transcripts from a small gene family encoding a specialized acyl-CoA desaturase. The presence of Kennedy pathway acyl transferase transcripts (LPAT and DGAT) and absence of alternative enzymes involved in TAG assembly such as lysophosphatidyl-chloline acyl transferase (LPCAT), suggests that the classical acyl-CoA dependent pathway is predominant in meadowfoam. This study located lipid and glucosinolate metabolic genes on the meadowfoam genetic map, and uncovered gene-specific SSCP and SNP polymorphisms that can be used in targeted molecular breeding to enhance this new oilseed crop.

## Supplemental Information

10.7717/peerj.915/supp-1 Figure S1 Single strand conformational polymorphism patterns used to map candidate genes

10.7717/peerj.915/supp-2 Table S1 Lookup table to interconvert between GenBank and TIGR meadowfoam sequence identifiers

10.7717/peerj.915/supp-3 Table S2 EST sequences comprising 1,352 meadowfoam contigs

10.7717/peerj.915/supp-4 Table S3 Meadowfoam SSRs mined from EST and methylation-filtered genomic libraries: repeat motifs, primer sequences, and reference sequence identifiers

10.7717/peerj.915/supp-5 Table S4 Primer sequences used in candidate gene mapping

10.7717/peerj.915/supp-6 Table S5 Meadowfoam SSRs mined from EST and methylation-filtered genomic libraries: SSR attributes and allele sizes detected in four meadowfoam genotypes

## Additional Information and Declarations

### DNA Deposition

The following information was supplied regarding the deposition of DNA sequences: GenBank, GI 166344198 through GI 166359570.
