# Supplementary figures and images for: Genes affecting novel seed constituents in Limnanthes alba Benth: transcriptome analysis of developing embryos and a new genetic map of meadowfoam
Source: PeerJ. 2015 May 19;3:e915. doi: 10.7717/peerj.915 (PMC4451031; doi:10.7717/peerj.915)

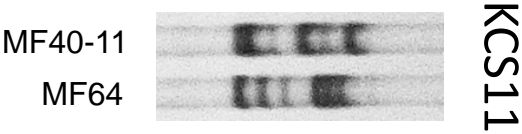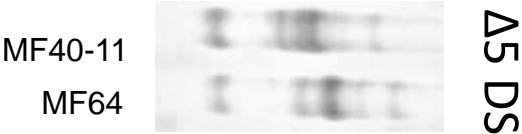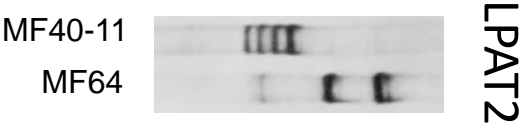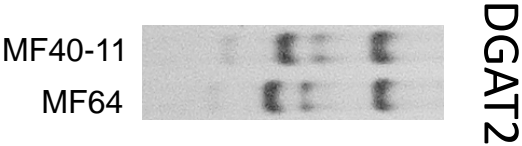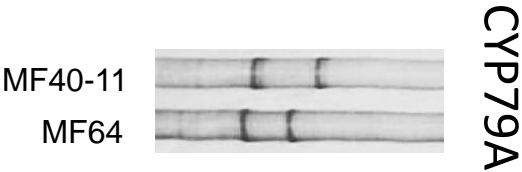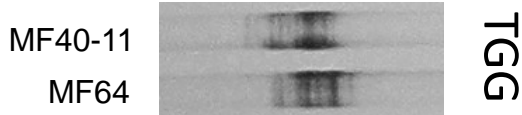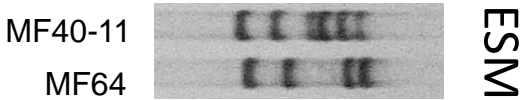

Supplement: Figure S1 [file peerj-03-915-s001.pdf]
